# Collargolum

**Published:** 1907-12

**Authors:** 


### CORRESPONDENCE

### Collargolum

**The Method of Action of Collargolum — Favors Leukocytosis — Interesting Letter from Schering & Glatz.**

*To the Editor of The Buffalo Medical Journal.*

Sir—Bacteriological investigations published of late have called renewed attention to the acknowledged fact that, in vitro, collargolum has no such bactericide powers as silver salts—such as the nitrate. As this fact has led to a priori denials of its clinical efficacy, permit us to point out that the indubitable therapeutic action of the remedy—demonstrated by almost four hundred publications—is not dependent on germicide properties.

By reason of its colloidal nature, collargolum has vigorous catalytic effects which induce or enhance the processes of oxidising bacterial toxins in the organism. This fact was proved experimentally by Schade, Hamburger, Robin and others. Professor Solis-Cohen (*Journ. Amer. Med. Asso.*, October 20, 1906,) stated:

> It is quite probable that the therapeutic value of colloidal silver is largely due to catalytic action in taking up and again yielding oxygen, thus destroying toxins, bacteria or diseased cells—a chemical amboceptor action, to take an illustration made familiar by Ehrlich—and through such action it may prevent or retard sepsis. It certainly has a definite therapeutic action and should be employed more extensively in larger and more frequent doses.

An exhaustive study of the leukocytogenic action of collargolum was recently published by Dunger (*Archiv f. klin. Medizin*, 1907, Vol. 91, No. 3-4). He found that an intravenous collargolum injection was immediately followed by hypoleukocytosis. One or two hours later a hyperleukocytosis always occurred, usually up to 130 to 150 per cent. with a maximum of 260 per cent. After twenty to twenty-four hours the number sank to its original figure. He explains the hypoleukocytosis as due to the destruction of neutrophiles, the later rise being an over compensation of the defect from the bone marrow. The collargolum leukocytosis is favorable because of the phagocytosis by which microörganisms are taken up and destroyed, and inorganic bodies such as silver particles are carried outside the blood current, as to joint cavities. The marrow irritation occasions a stimulus to the formation of immunising bodies. The leukocytic decomposition, setting free proteolytic ferment, is probably of the greatest importance for the solution and resorption of inflammatory exudations, especially of pneumonic infiltrations. This is confirmed by the author's experience with collargolum in a series of pneumonias.

That collargolum stimulates the formation of leukocytes, especially of the large, multinuclear forms, was also demonstrated by Rodsewicz, Achard and Weil, Ceresole, French, Widal and others.

When done under conditions closely simulating a clinical sepsis, animal experimentation with collargolum has always given favorable results, as the reports of many veterinary surgeons on the successful use of collargolum in infected horses, cows, etc., show. An artificially produced infection is, however, not analagous to one which occurs naturally, the intravenous injection of highly virulent bacterial cultures is far more brusque than the gradual development of a clinical sepsis. Moreover, in all laboratory experiments in which collargolum was administered to the animals simultaneously with the infectious material a negative result was to be expected, since the remedy is rapidly eliminated and had thus passed out of the body when the infection reached its height. Experimenters who waited with the administration of collargolum until the animals showed violent signs of illness, saved them (Beyer, Pinto and others).

In view of the importance of the subject, we trust the above will be accorded space in your esteemed columns.

Yours respectfully,

New York, November 18, 1907. SCHERING & GLATZ.

